# An analytic model for accurate spring constant calibration of rectangular atomic force microscope cantilevers

**DOI:** 10.1038/srep15828

**Published:** 2015-10-29

**Authors:** Rui Li, Hongfei Ye, Weisheng Zhang, Guojun Ma, Yewang Su

**Affiliations:** 1 State Key Laboratory of Structural Analysis for Industrial Equipment, Department of Engineering Mechanics, Dalian University of Technology, Dalian 116024, China; 2 State Key Laboratory of Digital Manufacturing Equipment & Technology, Huazhong University of Science and Technology, Wuhan 430074, China; 3 State Key Laboratory of Nonlinear Mechanics, Institute of Mechanics, Chinese Academy of Sciences, Beijing 100190, China; 4 Department of Civil and Environmental Engineering and Department of Mechanical Engineering, Northwestern University, Evanston, IL 60208, USA

## Abstract

Spring constant calibration of the atomic force microscope (AFM) cantilever is of fundamental importance for quantifying the force between the AFM cantilever tip and the sample. The calibration within the framework of thin plate theory undoubtedly has a higher accuracy and broader scope than that within the well-established beam theory. However, thin plate theory-based accurate analytic determination of the constant has been perceived as an extremely difficult issue. In this paper, we implement the thin plate theory-based analytic modeling for the static behavior of rectangular AFM cantilevers, which reveals that the three-dimensional effect and Poisson effect play important roles in accurate determination of the spring constants. A quantitative scaling law is found that the normalized spring constant depends only on the Poisson’s ratio, normalized dimension and normalized load coordinate. Both the literature and our refined finite element model validate the present results. The developed model is expected to serve as the benchmark for accurate calibration of rectangular AFM cantilevers.

Spring constant calibration of rectangular atomic force microscope (AFM) cantilevers is of fundamental importance in the measurement of pico/nano-Newton scale forces by an AFM with applications to many emerging technologies such as atomic manipulation^1^, imaging of molecules with atomic resolution^2^, characterization of complex mechanical properties^3^ and single asperity measurement^4^. However, the nominal spring constants are often not accurate and the manufacturers can only offer a wide range of their values^5^. Although the spring constant measurement is not a simple task, several widely used static or dynamic experimental calibration methods have been developed such as the static mass hanging method^6^, reference cantilever/spring method^7^,^8^,^9^, dynamic mass attachment method^10^, resonant frequency method^11^, and thermal noise method^12^. Very recently, some variants of the above-mentioned calibration methods have been proposed such as the Sader method for surface modified cantilevers^13^, direct thermal noise method for colloidal probe cantilevers^14^, calibration structures-based method^15^ and microchannel-aided method^16^. Some closely related topics also attract attention such as the nanoscale-resolved elasticity^17^ and the effect of surface stress on the stiffness of micro/nano cantilever^18^, which helps to gain insight into the measurement interpretation. It should be pointed out that there is the commonly used theoretical dimensional method^19^ which is based on the beam theory or derived from the plate theory with just approximate or numerical solutions^20^,^21^.

According to the dimensional method, the beam theory-based equation of spring constant for a rectangular cantilever is^19^


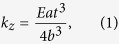


where *E* is the Young’s modulus of the material, 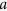, 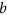 and *t* are the width, length and thickness of the cantilever, respectively. It is well known that equation (1) ignores the bowing of the cantilever across the width thus is only applicable to the cantilevers with 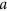
* << b*. For relatively wide cantilevers, the plate theory should be used, instead of the beam theory, to obtain more accurate results. However, the accurate analytic solution to the governing equation of a rectangular cantilever plate was extremely difficult to obtain due to the complexity of the mathematical model. Accordingly, the approximate/numerical solutions had to be developed to calibrate the AFM spring constants on a case-by-case basis. Obviously, the analytic solutions are necessary to capture the essence of the problem by quantitatively realizing the relation among the key parameters/quantities, which cannot be realized by an approximate/numerical solution.

According to the classical Kirchhoff thin plate theory^22^, the governing equation for the static problem of a thin plate is


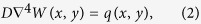


where 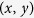 denote the coordinates in the plane where the plate lies, 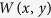 is the transverse deflection of the plate mid-plane, 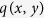 is the distributed transverse load, and 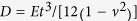 is the plate flexural stiffness in which 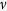 is the Poisson’s ratio. Using the classical methods, analytic solution of equation (2) can only be obtained for a rectangular plate with at least a pair of opposite edges simply supported. For the rectangular AFM cantilever which is modeled as the plate with one clamped edge and three free edges, i.e. the cantilever plate, the accurate analytic modeling was unavailable. Urgent need to address this issue motivates the present work.

We examine the mechanical behavior of the rectangular AFM cantilever by constructing the Hamiltonian variational principle from the original Hellinger-Reissner variational principle for the thin plate bending problem. The corresponding Hamiltonian system-based governing matrix equation is derived (see supplementary information for details) as


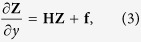


herein 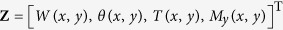, 
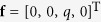
, 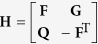 in which 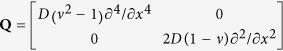, 
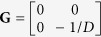
, and 
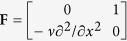
; 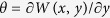, 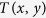 is the opposite of equivalent shear force 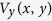, and 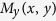 is the bending moment. Observing 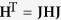, where 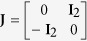 is the symplectic matrix in which 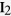 is 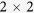 unit matrix, 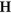 is a Hamiltonian operator matrix^23^ thus equation (3) is the Hamiltonian system-based governing equation for thin plate bending.

We develop an up-to-date superposition method^24^ to offer a rational way to accurately derive the analytic solution of the rectangular AFM cantilever with the length 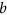 and width 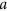 under a point load *P* at 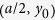, as illustrated in Fig. 1. The solution of the normalized load-point deflection 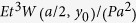 is obtained and denoted by the non-dimensional function 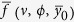, which is explicitly shown as


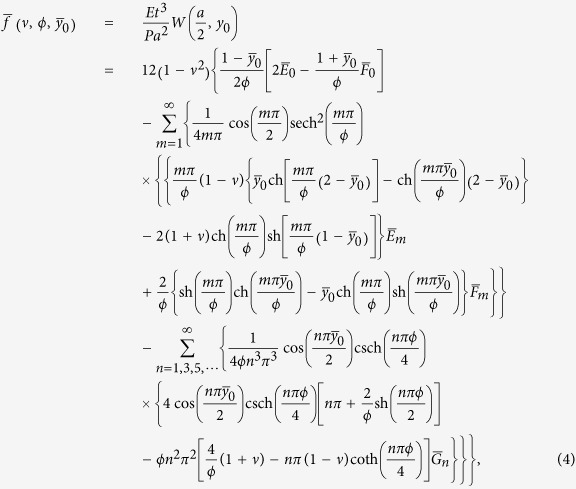


where 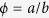 and 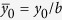, in which 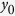 is the distance of load point away from the free end tip and it can range from 0 to *b*. The normalized constants 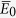, 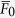, 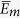, 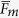 and 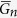 are determined by equations (S22)-(S26) of supplementary information.

We thus find a scaling law in determining the spring constants 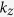:


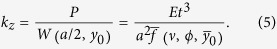


Equation (5) clearly shows that the spring constant 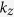, normalized by 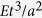, depends only on the Poisson’s ratio 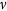, normalized dimension 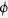 and normalized load coordinate 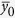. To illustrate this dependence, 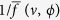 for an end-tip-loaded rectangular cantilever (i.e. 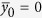) is plotted versus 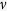 and 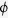 for different 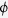 and 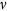, respectively, as shown in Fig. 2a,b.

Our finding is to be validated by the well-accepted finite element method (FEM). The normalized load-point deflections 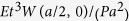 are tabulated in Table 1 for a rectangular cantilever with the aspect ratio 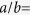  1/5, 2/9, 1/4, 2/7, 1/3, 2/5, 1/2, 2/3, 1, and 2, respectively, and the Poisson’s ratio 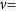 0, 0.25 and 0.4, respectively. Comparison is shown with FEM by ABAQUS software package where the 4-node general-purpose shell element S4R and uniform mesh with grid size 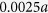 are employed. The number of terms for the present series solution is taken such that the results converge up to the last significant figure of four. It is evident in Table 1 that our analytic solutions agree perfectly with those by FEM which are regarded as the benchmarks in view of the absence of comparable analytic solutions, and our plate theory-based results give obvious accuracy improvement on the beam theory^25^. It is noted that the approximate methods had to be developed in the past when the theoretical analysis was needed^20^, which, however, cannot yield the results as accurate as presented here. It should be pointed out that the case with 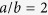 in Table 1 is rarely encountered in practice, but we still present the results in order to demonstrate the better accuracy as well as the broader applicability of the plate theory which better describes the behavior of the cantilevers at any scales, i.e. at the scales which are not merely restricted to the AFM cantilevers. The error variations of beam theory with 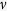 and 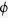 for different 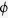 and 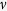, respectively, are plotted in Fig. 3a,b so that one can quickly assess what situations might yield a significant correction. It is interesting to observe in both Table 1 and Fig. 3 that there are very small errors for the cantilevers with 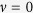 under relatively lower 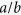. To explain this observation as well as the mechanism of accuracy improvement of our model, we would like to interpret more on the difference between the two theories. From the physical point of view, for the cantilever as depicted in Fig. 1, the beam theory actually describes such a cantilever plate with zero Poisson’s ratio: the uniformly distributed line load with the intensity of 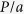 is applied along 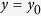 and no constraint is imposed in the 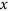 direction while no rotation around the 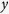 axis is allowed for the edges 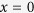 and 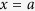. This could be rigorously proved from the mathematical point of view. The analytic solution of the normalized load-point deflection for such a specific plate is obtained by solving the governing equation 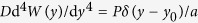, with the boundary conditions 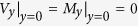 and 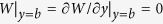 imposed, where 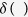 is the Dirac delta function; thus we can obtain


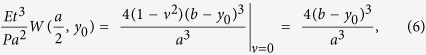


which reduces to the beam model solution. Therefore, compared with the three-dimensional model by the plate theory which incorporates the Poisson effect, the beam theory yields a much more simplified plane model and ignores the Poisson effect, which is numerically revealed in Table 1 by the independence of the beam theory-based results on 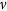 under the same 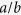. The ability of the plate theory to depict the field distribution of mechanical quantities over a cantilever is clearly reflected in Fig. 4a by comparison with the beam theory in Fig. 4b. Furthermore, it is seen from both Table 1 and Fig. 3b that the plate theory would predict either an increase or a decrease in the spring constant over the beam theory, but actually there is the important insight to be drawn that the plate theory always predicts a decrease in the spring constant over the beam theory when the Poisson ratio 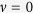. This can be explained by a simple inference. As illustrated in Fig. 5, for a cantilever with 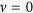, the plate model under a point load *P* at the central line (Fig. 5a) gives a lower spring constant than the same plate under a uniformly distributed line load with the intensity of 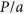 (Fig. 5b), because the former deflection at the load point is definitely large than the latter at the same point. On the other hand, this latter model (Fig. 5b) yields a lower spring constant than the same model plus the constraint that no rotation around the two side edges is allowed (Fig. 5c), because adding the constraint would enhance the stiffness. We have shown that the plate model in Fig. 5c is equal to the beam model under the same point load *P* as in Fig. 5a (see Fig. 5d). Therefore, if the spring constants of Fig. 5a–d are denoted by 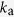, 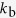, 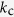, and 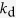, respectively, we conclude that 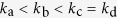.

We use the developed solutions to calibrate the spring constants for real cantilevers. Three sets of end-tip-loaded rectangular cantilevers, fabricated from the Perspex^21^ (PMMA, Young’s modulus 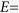  3 GPa, Poisson’s ratio 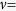  0.35), with the length 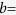 20 cm, thickness 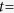  3 mm and width 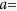  9.31, 6.53 and 3.29 cm, respectively, are considered. As shown in Table 2, the normalized spring constants 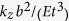 by the present analytic solution agree very well with the numerical results from ref. 21. We also examine several commercial rectangular AFM cantilevers in Table 3, with 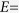  169 GPa and 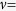  0.408^26^, of which the dimensions 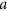, 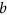 and 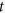 are listed in the table. We emphasize that the significant accuracy improvement (e.g. 5.7%) can be achieved by the present model even though the cantilevers tend to those with 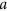
*<<b*.

In conclusion, we have explored an analytic approach to accurate spring constant calibration of rectangular AFM cantilevers based on the thin plate theory, by which the importance of the three-dimensional effect as well as Poisson effect is confirmed. The obtained solutions eliminate the errors caused by the classical beam theory, and hold for rectangular cantilevers with any aspect ratio 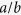. Deformation of an AFM cantilever involves seven load, material and geometry quantities: the point load 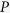, Poisson’s ratio 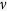, Young’s modulus 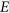, length 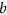, width 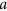, thickness 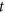 and load position 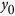. The scaling law in equation (5), verified by FEM, shows that the normalized spring constant depends only on three normalized quantities, i.e., 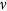, 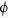 and 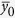. This scaling law could serve as the theoretical basis for analytically calibrating the spring constants of rectangular AFM cantilevers. It should be noted that in some applications the AFM cantilevers are coated with three-dimensional layers, and the influence of these layers on the spring constant calibration is not negligible. That is especially important for the applications that need to collect currents simultaneously, such as the conductive AFM^27^,^28^,^29^. One possible simple treatment is using the equivalent stiffness which appropriately incorporates the effect of the layers^30^. Our ongoing work is to obtain accurate enough equivalent stiffness so that the present model is applicable to the cantilevers with layers as well.

## Additional Information

**How to cite this article**: Li, R. *et al.* An analytic model for accurate spring constant calibration of rectangular atomic force microscope cantilevers. *Sci. Rep.* **5**, 15828; doi: 10.1038/srep15828 (2015).

## Supplementary Material

Supplementary Information

## Figures and Tables

**Figure 1:**
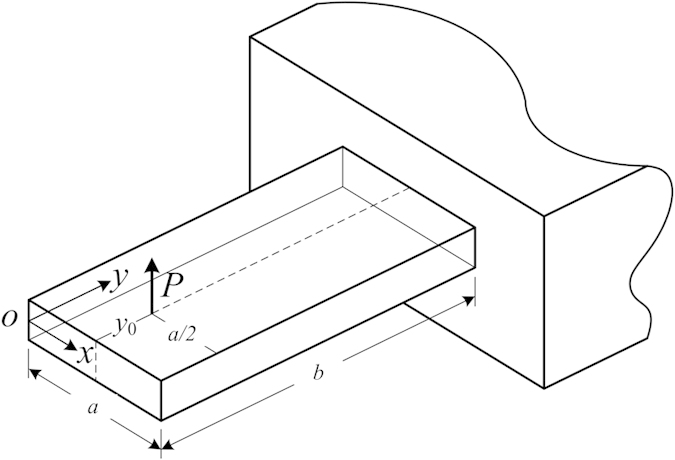
Theoretical model of a rectangular AFM cantilever under a point load.

**Figure 2:**
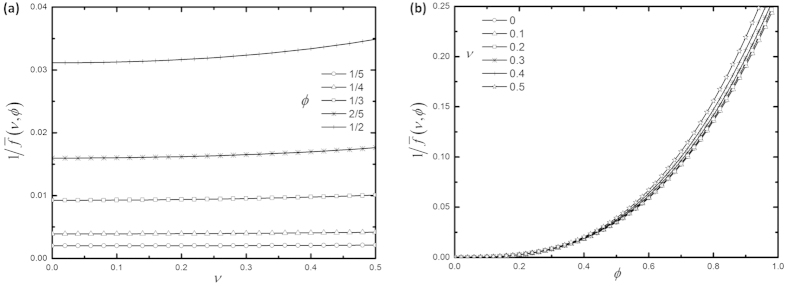
(**a**) 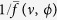 vs. 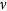 for different 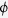, and **(b)** 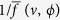 vs. 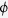 for different 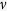 of an end-tip-loaded rectangular cantilever.

**Figure 3:**
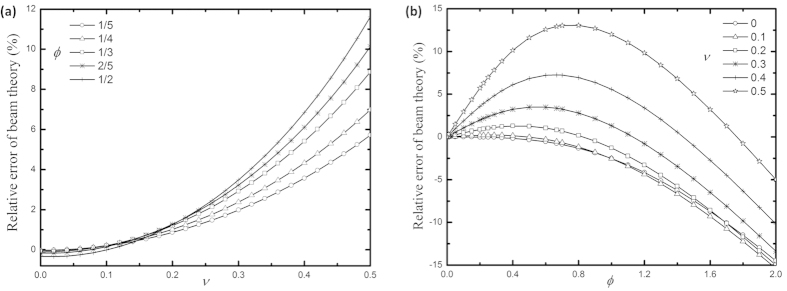
(**a**) Relative error of beam theory vs. 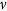 for different 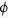, and **(b)** the error vs. 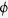 for different 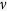 of an end-tip-loaded rectangular cantilever.

**Figure 4:**
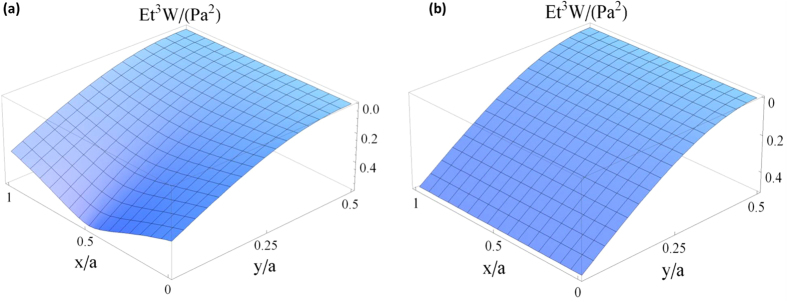
Comparison of plate and beam theories via three-dimensional plot of normalized deflection for an end-tip-loaded rectangular micro cantilever with *v* = 0.25 and a/b = 2. **(a)** Current analytic results from plate theory. **(b)** Results from beam theory.

**Figure 5:**
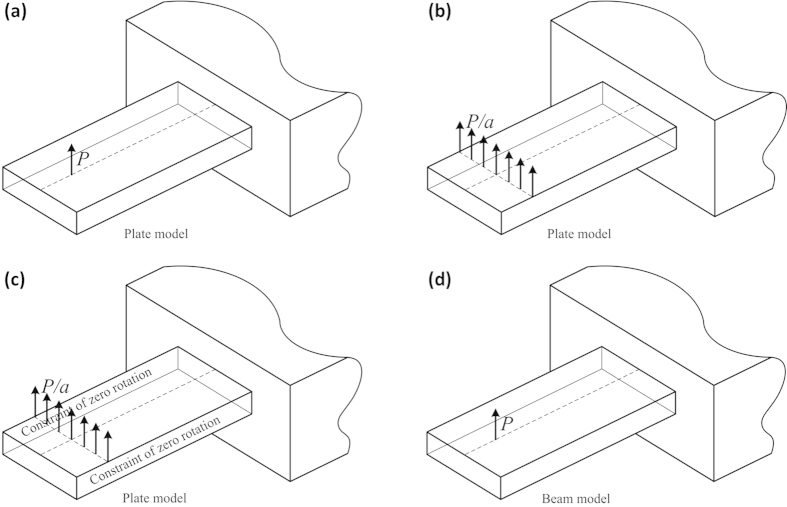
Schematic diagram for comparison of the spring constant between the plate theory and beam theory for a cantilever with *v* = 0. **(a)** The plate model under a point load *P*. **(b)** The plate model under a uniformly distributed line load with the intensity of 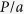. **(c)** The constrained plate model under the same load as in **(b)**. **(d)** The beam model under the same point load P as in **(a)**.

**Table 1:** Normalized load-point deflections of a rectangular cantilever with the point load *P* applied at the end tip (a/2,0).

*a/b*	*v*	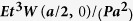			
		FEM	Present	Beam theory*	Error of beam theory
1/5	0	500.1	500.1	500	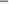 0.020%
	0.25	493.3	493.3	500	1.4%
	0.4	482.8	482.8	500	3.6%
2/9	0	364.6	364.6	364.5	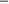 0.027%
	0.25	359.2	359.2	364.5	1.5%
	0.4	350.8	350.8	364.5	3.9%
1/4	0	256.1	256.1	256	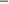 0.039%
	0.25	251.9	251.9	256	1.6%
	0.4	245.4	245.4	256	4.3%
2/7	0	171.6	171.6	171.5	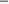 0.058%
	0.25	168.5	168.5	171.5	1.8%
	0.4	163.6	163.6	171.5	4.8%
1/3	0	108.1	108.1	108	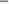 0.093%
	0.25	105.9	105.9	108	2.0%
	0.4	102.5	102.5	108	5.4%
2/5	0	62.61	62.61	62.5	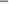 0.18%
	0.25	61.19	61.19	62.5	2.1%
	0.4	58.90	58.90	62.5	6.1%
1/2	0	32.11	32.11	32	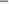 0.34%
	0.25	31.30	31.30	32	2.2%
	0.4	29.95	29.95	32	6.8%
2/3	0	13.61	13.61	13.5	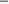 0.81%
	0.25	13.24	13.24	13.5	2.0%
	0.4	12.59	12.59	13.5	7.2%
1	0	4.103	4.103	4	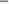 2.5%
	0.25	4.007	4.007	4	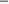 0.17%
	0.4	3.789	3.789	4	5.6%
2	0	0.5847	0.5847	0.5	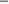 14%
	0.25	0.5838	0.5838	0.5	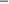 14%
	0.4	0.5567	0.5567	0.5	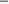 10%

^*^The beam theory-based normalized deflection^25^ 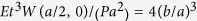.

**Table 2:** Normalized spring constants of several end-tip-loaded rectangular cantilevers fabricated from Perspex.

No.	Cantilever size	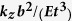	
		Ref. 21	Present
1	b = 20 cm, t = 3 mm, a = 9.31 cm	0.12	0.1221
2	b = 20 cm, t = 3 mm, a = 6.53 cm	0.085	0.08489
3	b = 20 cm, t = 3 mm, a = 3.29 cm	0.042	0.04206

**Table 3:** Spring constants of several commercial rectangular cantilevers at end tips.

No.	Cantilever type	*K*_*z*_ (N/m)		
		Present	Ref. 26	Error of Ref. 26
1	Nanosensors PPP-NCCR-50 silicon tapping mode			
	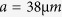 ; 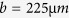 ; 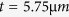	27.65	26.8	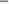 3.1%
2	Olympus OMCL-RC800PSA-1 Si_3_N_4_ contact mode			
	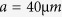 ; 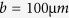 ; 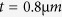	0.9207	0.87	−5.5%
	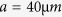 ; 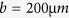 ; 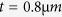	0.1122	0.11	−2.0%
3	Nanosensors silicon extra tall tips			
	SD-PXL-FM 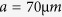 ; 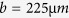 ; 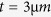	7.388	7	−5.3%
	SD-PXL-CON 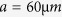 ; 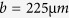 ; 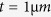	0.2332	0.22	−5.7%
