# Supplementary material for: An analytic model for accurate spring constant calibration of rectangular atomic force microscope cantilevers
Source: Sci Rep. 2015 Oct 29;5:15828. doi: 10.1038/srep15828 (PMC4625185; doi:10.1038/srep15828)
Supplement: Supplementary Information [file srep15828-s1.doc]

**Supplementary Information for “An analytic model for accurate spring constant calibration of rectangular atomic force microscope cantilevers”**

Rui Li[[1]](#footnote-2),2, Hongfei Ye^1^, Weisheng Zhang^1^, Guojun Ma^1^ & Yewang Su^3,4^

In this file, we elaborate on the solution of governing equation for static problem of a rectangular AFM cantilever, by which we find the analytic scaling law [equation (5) of the main text] for spring constant calibration.

**S.1. Governing equation within the framework of Hamiltonian system**

The Hellinger-Reissner variational principle^S1^ for bending of a thin plate is

$\delta\Pi_{H-R}=0$, (S1)

where the generalized potential energy functional with two kinds of variables is

$\begin{matrix} \Pi_{H-R}=\iint_{\Omega} \left\{-M_{x}\frac{\partial^{2}W}{\partial x^{2}}-2M_{xy}\frac{\partial^{2}W}{\partial x\partial y}-M_{y}\frac{\partial^{2}W}{\partial y^{2}}\right. \\ \left.-\frac{1}{2D\left(1-\nu^{2}\right)}\left[M_{x}^{2}+M_{y}^{2}-2\nu M_{x}M_{y}+2\left(1+\nu\right)M_{xy}^{2}\right]-qW\right\}\text{d}x\text{d}y \end{matrix}$, (S2)

herein the surface integral is taken over the domain $\Omega$ and the line integrals with respect to boundary conditions are not taken into account. $q$ is the distributed transverse load, $\nu$ is the Poisson’s ratio, $D$ is the flexural rigidity, $W$ is the transverse deflection of the plate mid-plane, $M_{x}$ and $M_{y}$ are the bending moments, and $M_{xy}$ is the torsional moment. Assuming the independence of $M_{x}$, $M_{y}$, $M_{xy}$ and $W$ and the arbitrariness of their variations, equation (S1) gives the natural conditions of stationary $\Pi_{H-R}$, two of which are $M_{x}=-D\left(\partial^{2}W/\partial x^{2}+\nu\partial^{2}W/\partial y^{2}\right)$ and $M_{xy}=-D\left(1-\nu\right)\partial^{2}W/\partial x\partial y$. Substituting them into equation (S2), we have a new functional $\Pi^{*}$:

$\begin{matrix} \Pi^{*}=\iint_{\Omega} \left[\frac{D}{2}\left(\frac{\partial^{2}W}{\partial x^{2}}\right)^{2}+\frac{D}{2}\left(\frac{\partial^{2}W}{\partial y^{2}}\right)^{2}+D\left(1-\nu\right)\left(\frac{\partial^{2}W}{\partial x\partial y}\right)^{2}\right. \\ \left.+D\nu\frac{\partial^{2}W}{\partial x^{2}}\frac{\partial^{2}W}{\partial y^{2}}-\frac{D}{2\left(1-\nu^{2}\right)}\left(\frac{M_{y}}{D}+\frac{\partial^{2}W}{\partial y^{2}}+\nu\frac{\partial^{2}W}{\partial x^{2}}\right)^{2}-qW\right]\text{d}x\text{d}y \end{matrix}$. (S3)

Define

$\theta=\frac{\partial W}{\partial y}$ (S4)

as well as the Lagrange multiplier $T$, $\Pi^{*}$ is transformed into $\Pi_{H}$:

$\begin{matrix} \Pi_{H}=\iint_{\Omega} \left[\frac{D}{2}\left(\frac{\partial^{2}W}{\partial x^{2}}\right)^{2}+\frac{D}{2}\left(\frac{\partial\theta}{\partial y}\right)^{2}+D\nu\frac{\partial^{2}W}{\partial x^{2}}\frac{\partial\theta}{\partial y}+D\left(1-\nu\right)\left(\frac{\partial\theta}{\partial x}\right)^{2}\right. \\ \left.-\frac{D}{2\left(1-\nu^{2}\right)}\left(\frac{M_{y}}{D}+\frac{\partial\theta}{\partial y}+\nu\frac{\partial^{2}W}{\partial x^{2}}\right)^{2}+T\left(\theta-\frac{\partial W}{\partial y}\right)-qW\right]\text{d}x\text{d}y \end{matrix}$. (S5)

Noting the arbitrariness of $\delta T$, $\delta M_{y}$, $\delta W$ and $\delta\theta$,

$\delta\Pi_{H}=0$ (S6)

yields equation (3) of the main text, i.e.,

$\frac{\partial\mathbf{Z}}{\partial y}=\mathbf{HZ}+\mathbf{f}$, (S7)

where $\mathbf{H}=\left[\begin{matrix} \mathbf{F} & \mathbf{G} \\ \mathbf{Q} & -\mathbf{F}^{\text{T}} \end{matrix}\right]$, $\mathbf{Q}=\left[\begin{matrix} D\left(\nu^{2}-1\right)\partial^{4}/\partial x^{4} & 0 \\ 0 & 2D\left(1-\nu\right)\partial^{2}/\partial x^{2} \end{matrix}\right]$, $\mathbf{F}=\left[\begin{matrix} 0 & 1 \\ -\nu\partial^{2}/\partial x^{2} & 0 \end{matrix}\right]$, $\mathbf{G}=\left[\begin{matrix} 0 & 0 \\ 0 & -1/D \end{matrix}\right]$, $\mathbf{Z}=\left[w,\theta,T,M_{y}\right]^{\text{T}}$, and $\mathbf{f}=\left[0,0,q,0\right]^{\text{T}}$. $T=-V_{y}$ is inferred from equation (S7), where $V_{y}=-D\left[\partial^{3}w/\partial y^{3}+\left(2-\nu\right)\partial^{3}w/\partial x^{2}\partial y\right]$ is the equivalent shear force. It is noted that $\mathbf{H}$ is a Hamiltonian operator matrix^S1^ and equation (S7) is the governing equation for thin plate bending within the framework of Hamiltonian system.

**S.2. Analytic solution of rectangular cantilever under point load**

The problem (see Fig. 1 of the main text) is solved by superposing three sub-problems: (A) the plate is simply supported at the edge $y=b$ and slidingly clamped at the other three edges, with the point load *P* applied at $\left(a/2,y_{0}\right)$; (B) the plate is slidingly clamped at $x=0$ and $x=a$, with the slope $\left.\theta\right|_{y=0}=\sum_{m=0,1,2,\cdots}^{\infty} E_{m}\cos\left(m\pi x/a\right)$ and moment $\left.M_{y}\right|_{y=b}=\sum_{m=0,1,2,\cdots}^{\infty} F_{m}\cos\left(m\pi x/a\right)$ applied at the slidingly clamped edge $y=0$ and simply supported edge $y=b$, respectively; (C) the plate is slidingly clamped at $y=0$ and simply supported at $y=b$, with the slopes $\left.\theta\right|_{x=0}=\sum_{n=1,3,5,\cdots}^{\infty} G_{n}\cos\left[n\pi y/\left(2b\right)\right]$ and $\left.\theta\right|_{x=a}=-\sum_{n=1,3,5,\cdots}^{\infty} G_{n}\cos\left[n\pi y/\left(2b\right)\right]$ applied at the slidingly clamped edges $x=0$ and $x=a$, respectively. Here $E_{m}$, $F_{m}$ and $G_{n}$ are the Fourier expansion coefficients.

In the Hamiltonian system, the homogeneous equation of equation (S7)

$\frac{\partial\mathbf{Z}}{\partial y}=\mathbf{HZ}$ (S8)

could be handled by separation of variables^S2^, i.e., imposing

$\mathbf{Z}=\mathbf{X}\left(x\right) Y\left(y\right)$ (S9)

such that

$\begin{matrix} \frac{\text{d}Y\left(y\right)}{\text{d}y}=\mu Y\left(y\right) \\ \mathbf{HX}\left(x\right)=\mu\mathbf{X}\left(x\right) \end{matrix}$, (S10a,b)

where $\mu$ is the eigenvalue and $\mathbf{X}\left(x\right)$ is the corresponding eigenvector.

We begin with the plate with $x=0$ slidingly clamped and $x=a$ simply supported. The eigenvalue problem (S10b) and the boundary conditions

$\begin{matrix} \left.\frac{\text{d}W\left(x\right)}{\text{d}x}\right|_{x=0}=\left.V_{x}\left(x\right)\right|_{x=0}=0 \\ \left.W\left(x\right)\right|_{x=0}=\left.M_{x}\left(x\right)\right|_{x=a}=0 \end{matrix}$ (S11)

determine the eigenvalue equation $\cos^{2}\left(\mu a\right)=0$, where $V_{x}\left(x\right)$ is an x-component of equivalent shear force $V_{x}$. This gives the eigenvalues $\mu_{n}=n\pi/\left(2a\right)$ and $\mu_{-n}=-n\pi/\left(2a\right)$ for $n=1,3,5,\cdots$. The corresponding eigenvectors are

$\begin{matrix} \mathbf{X}_{n}^{0}\left(x\right)=\cos\left(\mu_{n}x\right)\left\{1, \mu_{n}, D\mu_{n}^{3}\left(\nu-1\right), D\mu_{n}^{2}\left(\nu-1\right)\right\}^{\text{T}} \\ \mathbf{X}_{n}^{1}\left(x\right)=\cos\left(\mu_{n}x\right)\left\{1, 1+\mu_{n}, D\mu_{n}^{2}\left[1+\nu-\mu_{n}\left(1-\nu\right)\right],-D\mu_{n}\left[2+\mu_{n}\left(1-\nu\right)\right] \right\}^{\text{T}} \\ \mathbf{X}_{-n}^{0}\left(x\right)=\cos\left(\mu_{n}x\right)\left\{1, -\mu_{n}, D\mu_{n}^{3}\left(1-\nu\right), D\mu_{n}^{2}\left(\nu-1\right)\right\}^{\text{T}} \\ \mathbf{X}_{-n}^{1}\left(x\right)=\cos\left(\mu_{n}x\right)\left\{\left(1+\mu_{n}\right)/\mu_{n}, -\mu_{n}, D\mu_{n}^{2}\left[2+\mu_{n}\left(1-\nu\right)\right],D\mu_{n}\left[1+\nu-\mu_{n}\left(1-\nu\right)\right]\right\}^{\text{T}} \end{matrix}$, (S12)

where $\mathbf{X}_{n}^{0}\left(x\right)$ and $\mathbf{X}_{n}^{1}\left(x\right)$ denote the zeroth-order and first-order eigenvectors of $\mu_{n}$ while $\mathbf{X}_{-n}^{0}\left(x\right)$ and $\mathbf{X}_{-n}^{1}\left(x\right)$ denote those of $\mu_{-n}$. The symplectic conjugacy and orthogonality^S2^ are satisfied between any two eigenvectors, i.e., $\int_{0}^{a} \mathbf{X}_{n}^{0}\left(x\right)^{\text{T}}\mathbf{JX}_{-n}^{1}\left(x\right)\text{d}x\neq0$ and $\int_{0}^{a} \mathbf{X}_{n}^{1}\left(x\right)^{\text{T}}\mathbf{JX}_{-n}^{0}\left(x\right)\text{d}x\neq0$ while any other combinations of two eigenvectors are orthogonal. The solution of equation (S7) is

$\mathbf{Z}=\mathbf{X}\left(x\right) \mathbf{Y}\left(y\right)$, (S13)

where

$\begin{matrix} \mathbf{X}\left(x\right)=\left[\cdots,\mathbf{X}_{n}^{0}\left(x\right),\mathbf{X}_{n}^{1}\left(x\right),\cdots,\mathbf{X}_{-n}^{1}\left(x\right),\mathbf{X}_{-n}^{0}\left(x\right),\cdots\right] \\ \mathbf{Y}\left(y\right)= \left[\cdots,Y_{n}^{0}\left(y\right),Y_{n}^{1}\left(y\right),\cdots,Y_{-n}^{1}\left(y\right),Y_{-n}^{0}\left(y\right),\cdots\right]^{\text{T}} \end{matrix}$. (S14)

Substituting equation (S13) into equation (S7) yields

$\frac{\text{d}\mathbf{Y}}{\text{d}y}-\mathbf{MY}=\mathbf{G}$, (S15)

where $\mathbf{M}=diag\left(\cdots,\mathbf{P}_{n},\cdots,\mathbf{Q}_{n},\cdots\right)$, $\mathbf{P}_{n}=\left[\begin{matrix} \mu_{n} & 1 \\ 0 & \mu_{n} \end{matrix}\right]$, $\mathbf{Q}_{n}=\left[\begin{matrix} -\mu_{n} & 0 \\ 1 & -\mu_{n} \end{matrix}\right]$, $\mathbf{G}=\left[\cdots,g_{n}^{0},g_{n}^{1},\cdots,g_{-n}^{1},g_{-n}^{0},\cdots\right]^{\text{T}}$ is the column matrix of expansion coefficients of $\mathbf{f}$. For the case of point loading at $\left(x_{0},b/2\right)$, we obtain

$\begin{matrix} g_{n}^{0}=-\frac{2Pa\left(2a+n\pi\right)}{Dn^{3}\pi^{3}}\cos\left(\frac{n\pi x_{0}}{2a}\right)\delta\left(y-\frac{b}{2}\right) \\ g_{n}^{1}=\frac{2Pa}{Dn^{2}\pi^{2}}\cos\left(\frac{n\pi x_{0}}{2a}\right)\delta\left(y-\frac{b}{2}\right) \\ g_{-n}^{0}=-\frac{2Pa}{Dn^{2}\pi^{2}}\cos\left(\frac{n\pi x_{0}}{2a}\right)\delta\left(y-\frac{b}{2}\right) \\ g_{-n}^{1}=\frac{2Pa}{Dn^{2}\pi^{2}}\cos\left(\frac{n\pi x_{0}}{2a}\right)\delta\left(y-\frac{b}{2}\right) \end{matrix}$, (S16)

where $\delta( )$ is the Dirac delta function. Substituting equations (S16) into equation (S15) gives

$\begin{matrix} Y_{n}^{0}\left(y\right)=\text{e}^{\frac{n\pi y}{2a}}\left(C_{n1}y+C_{n2}\right)-\frac{Pa}{Dn^{3}\pi^{3}}\text{e}^{-\frac{n\pi\left(b-2y\right)}{4a}}\cos\left(\frac{n\pi x_{0}}{2a}\right)\left[4a+n\pi\left(2+b-2y\right)\right]H\left(y-\frac{b}{2}\right) \\ Y_{n}^{1}\left(y\right)=C_{n1}\text{e}^{\frac{n\pi y}{2a}}+\frac{2Pa}{Dn^{2}\pi^{2}}\text{e}^{-\frac{n\pi\left(b-2y\right)}{4a}}\cos\left(\frac{n\pi x_{0}}{2a}\right)H\left(y-y_{0}\right) \\ Y_{-n}^{0}\left(y\right)=\text{e}^{-\frac{n\pi y}{2a}}\left(C_{n3}y+C_{n4}\right)-\frac{Pa}{Dn^{2}\pi^{2}}\text{e}^{\frac{n\pi\left(b-2y\right)}{4a}}\cos\left(\frac{n\pi x_{0}}{2a}\right)\left(2+b-2y\right)H\left(y-y_{0}\right) \\ Y_{-n}^{1}\left(y\right)=C_{n3}\text{e}^{-\frac{n\pi y}{2a}}+\frac{2Pa}{Dn^{2}\pi^{2}}\text{e}^{\frac{n\pi\left(b-2y\right)}{4a}}\cos\left(\frac{n\pi x_{0}}{2a}\right)H\left(y-y_{0}\right) \end{matrix}$, (S17)

where $H( )$ is the Heaviside theta function, $C_{n1}-C_{n4}$ are to be determined by imposing the remaining boundary conditions at $y=0$ and $y=b$. For the plate with $y=0$ and $y=b$ slidingly clamped, we have

$\left.\theta\right|_{y=0,b}=\left.T\right|_{y=0,b}=0$. (S18)

Substituting equations (S12) and (S17) into equations (S14) then equation (S13) and imposing equation (S18), $C_{n1}-C_{n4}$ are obtained. Thus we obtain the analytic solution of the plate with $x=a$ simply supported and the other three edges slidingly clamped under the point load *P* at $\left(x_{0},b/2\right)$. By conversion of coordinates, we obtain the analytic solution of the plate with $y=b$ simply supported and the other three edges slidingly clamped under the point load *P* at $\left(a/2,y_{0}\right)$. For convenience, we present the normalized load-point deflection solution as

, (S19)

where and $\bar{y}_{0}=y_{0}/b$.

By way of the similar procedure as stated above, we obtain the analytic solution of the plate with $x=0$ and $x=a$ slidingly clamped and the slope $\left.\theta\right|_{y=0}=\sum_{m=0,1,2,\cdots}^{\infty} E_{m}\cos\left(m\pi x/a\right)$ and moment $\left.M_{y}\right|_{y=b}=\sum_{m=0,1,2,\cdots}^{\infty} F_{m}\cos\left(m\pi x/a\right)$ applied at the slidingly clamped edge $y=0$ and simply supported edge $y=b$, respectively. The normalized deflection at $\left(a/2,y_{0}\right)$ is

. (S20)

Here and henceforth the normalized constants $\bar{E}_{\text{s}}=DE_{\text{s}}/\left(Pa\right)$, $\bar{F}_{\text{s}}=F_{\text{s}}/P$, and $\bar{G}_{\text{s}}=DG_{\text{s}}/\left(Pa\right)$, where the subscript “s” could be 0, *m*, *n*, or *i*.

For the plate slidingly clamped at $y=0$ and simply supported at $y=b$, with the slopes $\left.\theta\right|_{x=0}=\sum_{n=1,3,5,\cdots}^{\infty} G_{n}\cos\left[n\pi y/\left(2b\right)\right]$ and $\left.\theta\right|_{x=a}=-\sum_{n=1,3,5,\cdots}^{\infty} G_{n}\cos\left[n\pi y/\left(2b\right)\right]$ applied at the slidingly clamped edges $x=0$ and $x=a$, respectively, we obtain

.(S21)

Noting $W\left(a/2,y_{0}\right)=\sum_{i=1}^{3} W_{i}\left(a/2,y_{0}\right)$, the sum of equations (S19)-(S21) gives the explicit expression of the function of the main text, i.e. equation (4):

. (4)

The requirements of $M_{y}=0$ at $y=0$, $\theta=0$ at $y=b$ and $M_{x}=0$ at $x=0$ yield

, (S22)

, (S23)

(S24)

and

(S25)

for $i=1,2,3,\cdots$, and

(S26)

for $i=1,3,5,\cdots$. Equations (S22)-(S26) determine the normalized constants $\bar{E}_{\text{s}}$, $\bar{F}_{\text{s}}$ and $\bar{G}_{\text{s}}$ in equation (4). Accordingly, the scaling law is found, as shown in equation (5) of the main text.

**References**

S1. Yao, W., Zhong W. & Lim, C. W. *Symplectic elasticity* (World Scientific, 2009).

S2. Li, R., Zhong, Y. & Li, M. Analytic bending solutions of free rectangular thin plates resting on elastic foundations by a new symplectic superposition method. *Proc. R. Soc. A-Math. Phys. Eng. Sci.* **469**, 20120681 (2013).

1. State Key Laboratory of Structural Analysis for Industrial Equipment, Department of Engineering Mechanics, Dalian University of Technology, Dalian 116024, China. ^2^State Key Laboratory of Digital Manufacturing Equipment & Technology, Huazhong University of Science and Technology, Wuhan 430074, China. ^3^State Key Laboratory of Nonlinear Mechanics, Institute of Mechanics, Chinese Academy of Sciences, Beijing 100190, China. ^4^Department of Civil and Environmental Engineering and Department of Mechanical Engineering, Northwestern University, Evanston, IL 60208, USA. Correspondence and requests for materials should be addressed to R.L. (email: ruili@dlut.edu.cn) and Y.S. (email: yewangsu@imech.ac.cn) [↑](#footnote-ref-2)
